# Modifying laboratory testing via home brew during the COVID-19 pandemic

**DOI:** 10.1017/cts.2021.5

**Published:** 2021-01-25

**Authors:** Jeffery H. Moran, Larry Kessler, Jennifer Moylan, Craig Forrest, Karl Boehme, Josh Kennedy, Alex Greninger, Geoff Baird, Ericka Olgaard, Laura James

**Affiliations:** 1 Departments of Pharmacology and Toxicology, Microbiology, Pathology, and Pediatrics, College of Medicine, University of Arkansas for Medical Sciences, Little Rock, Arkansas, USA; 2 Department of Health Services, School of Public Health, University of Washington, Seattle, WA, USA; 3 Department of Physiology, College of Medicine, University of Kentucky, Lexington, Kentucky, USA; 4 Department of Laboratory Medicine and Pathology, School of Medicine, University of Washington, Seattle, Washington, USA

**Keywords:** Diagnostic tests, COVID-19, laboratory developed tests

## Abstract

Rapid development and deployment of diagnostic testing for COVID-19 have been a key component of the public health response to the pandemic. Out of necessity, academic and other clinical laboratories developed laboratory testing innovations for COVID-19 to meet clinical testing demands. In addition to constraints on local testing supplies and equipment, a rapidly changing regulatory framework created challenges for translational scientists. Illustrative examples of approaches used to develop laboratory tests during the early stages of the COVID-19 pandemic demonstrate effective team science approaches to this challenging clinical care and public health emergency. These experiences and the associated lessons learned are relevant to the development of public health response plans for future pandemics.

## Introduction

The first United States (US) COVID-19 case occurred on January 20, 2020 and involved an individual from Seattle, Washington, who had recently traveled to Wuhan, China [1]. The case was confirmed by a test kit developed by the US Centers for Disease Control (CDC). Over the following weeks and months, a number of laboratory developed tests (LDTs) were developed by US academic centers and other clinical laboratories. LDTs, colloquially known as “home brew” or “in house” *in vitro* diagnostic tests, have been a subject of controversy among the US Food and Drug Administration (FDA) and clinical testing laboratories for years [2,3]. By January 31, 2020, a US public health emergency was declared, and the FDA issued guidance to allow emergency use authorization (EUA) for laboratories and companies to develop and use these critical diagnostic tests to measure the extent of the pandemic as well as provide diagnoses for patients.

The CDC received the first EUA on February 4, 2020. Unfortunately, problems with the initial CDC test (e.g., contamination in negative control samples) slowed progress in test development over the subsequent weeks and months [4]. Collectively, the swiftly moving pandemic, the lack of clarity around regulatory roles, and the variable quality of developed tests hampered initial efforts to use diagnostic testing as a leading tool in the public health armamentarium. In addition, these events created both the need and opportunity for laboratory scientists to lead in the translation of bench science to clinical laboratory testing to meet public health demands.

The following manuscript describes the contributions of several academic laboratories, including clinical and research laboratories at academic health organizations, some of which are recipients of Clinical and Translational Science Awards (CTSAs), to the development of COVID-19-associated diagnostic tests. Interactions of these laboratories with state departments of health, and other state-associated laboratories, to create a state-based public health response to COVID-19 testing illustrate many components of the translational research process. These experiences provide clinical and translational researchers the opportunity to learn from other academic health organizations, to identify new partners for future research collaborations addressing public health responses to pandemics, and to potentially influence future diagnostic testing approaches.

## Laboratory Developed Tests (LDTs) prior to the COVID-19 Pandemic

Since the onset of the COVID-19 pandemic, three major categories of laboratory tests have emerged, two of which are generally considered to be diagnostic tests because of their ability to detect active infection. Briefly, reverse transcriptase-polymerase chain reaction (RT-PCR) is considered to be the gold standard to detect active infection. RT-PCR is highly sensitive and specific and is typically performed in highly specialized laboratories. The lack of availability of pre-analysis reagents during the early stages of the pandemic created testing delays in many areas of the United States (further discussed). Antigen tests, the second category of diagnostic tests detect viral proteins and are often configured as lateral flow or chemiluminescent immunoassays [5]. Antigen tests have lower sensitivity and higher rates of false positives compared with RT-PCR. The advantages of antigen tests include quicker reporting times and simpler testing formats that do not require trained laboratory professionals. Antigen tests are indicated for the testing of symptomatic individuals and have the potential to expand testing to hard-to-reach and rural *populations*. Serologic (antibody-based) assays, the third category, are important for addressing the adaptive immune response to SARS-CoV-2 infection and for epidemiologic studies [6]. Serologic assays indicate prior infection, are useful for understanding community spread and population susceptibility [7] and may be valuable for identifying COVID-19 associated complications, such as cardiovascular and neurologic sequelae, in patients who did not have prior RT-PCR or antigen-based testing to confirm diagnosis. These assays may also help our understanding of immunity to SARS-CoV-2 infection, including both acquired and vaccine-induced responses. Emerging data suggest that COVID-19 antibodies are detectable for three to four months or longer after infection [8, 9].

**Fig. 1.**
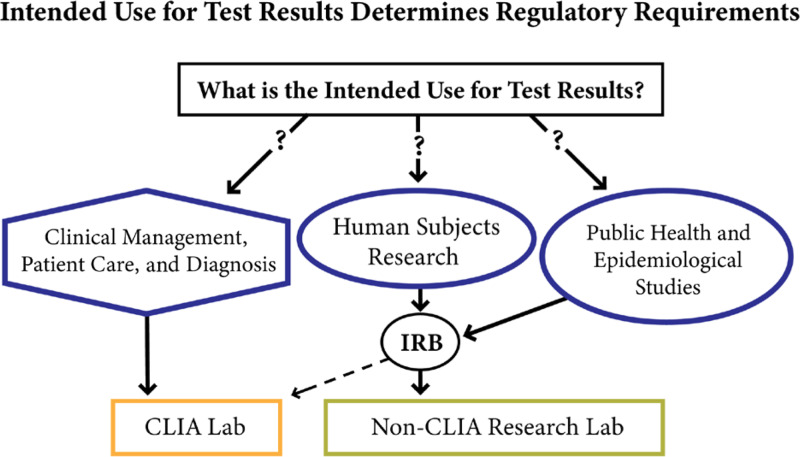
*Data use drives the process of choosing an appropriate laboratory for testing*. Clinical laboratory improvement amendments (CLIA)-certified laboratories report data used for clinical management, patient care, and diagnosis. Non-CLIA-certified laboratories can only report data for research and other public health investigations. Institutional Review Board (IRB) safeguards human subject research regardless of laboratory type.

Effective laboratory testing responses during the COVID-19 pandemic required the engagement of many stakeholders, all of whom were required to make real-time, data-driven decisions to respond to the health needs imposed by the pandemic. For example, clinicians, public health officials, and researchers require data to support (1) patient care and diagnosis, (2) research (basic or human subjects research), and (3) public health and epidemiological investigations. Reporting data in these situations is complex, and care must be taken to use certified laboratories and approved diagnostics when required by law. As depicted in Fig. 1, the intended use of test results determines the regulatory requirements for the development and use of laboratory tests. Without question, data used for COVID-19 patient care and diagnosis required that a laboratory possess a CLIA (1988 Clinical Laboratory Improvement Amendments) certificate. Data used for research or public health investigations, especially during emergencies, do not require the use of CLIA-certified laboratories. Data quality in these instances is typically guided by peer review, and in particular, human subjects research data are subject to institutional review board (IRB) regulations and oversight [10–12].

**Fig. 2.**
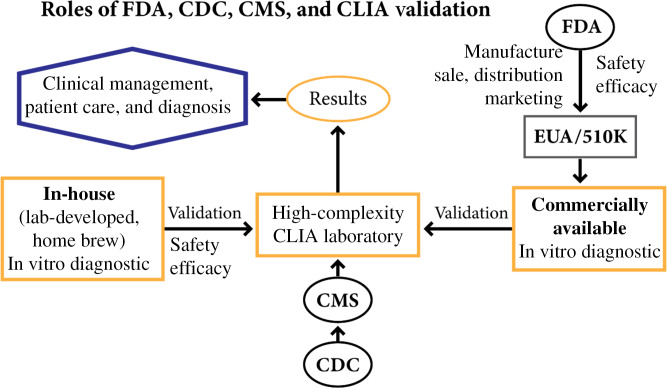
*The US Food and Drug Administration (FDA), Centers for Disease Control (CDC), Center for Medicare and Medicaid Services (CMS) each have defined roles in validation and use of both commercially available and in-house laboratory-developed tests.* COVID-19 testing for clinical care must be performed in clinical laboratory improvement amendments (CLIA) Laboratories certified for high-complexity testing. CMS administers requirements of CLIA validation while CDC provides the scientific infrastructure for CMS. The FDA ensures safety, effectiveness, and appropriate manufacture of commercially available tests. EUA, emergency use authorization.

Not only do COVID-19 researchers, especially those developing validated tests, need to understand the nuance of data reporting, it is also critical that they understand regulations for laboratory diagnostic tests. All commercially marketed laboratory diagnostic tests are regulated as medical devices under FDA oversight. However, in the COVID-19 pandemic, nimble researchers began developing tests within individual laboratories for local testing (i.e., LDTs). As with commercially available tests, these LDTs were subject to CLIA regulations (Fig. 2). On January 31, 2020, the US Department of Health and Human Services (HHS) declared a public health emergency due to the coronavirus, which was retroactive to January 27, 2020 [13]. Subsequently, the FDA asserted its authority over COVID-19 testing by announcing on February 29, 2020:

The immediately in effect guidance issued today describes the circumstances where the FDA does not intend to object to the use of these tests for clinical testing while the laboratories are pursuing an EUA with the FDA. Importantly, this policy only applies to laboratories that are certified to perform high-complexity testing consistent with requirements under Clinical Laboratory Improvement Amendments.

The FDA’s announcement caused a delay in some instances at the state level (described further), in that laboratories were able to provide validated testing for patient diagnosis, but were unable to get EUA authorization to do so. Eventually, the HHS rescinded the FDA’s authority on August 20, 2020, allowing LDTs to be used for any purpose, including generating results for clinical management, provided CLIA requirements had been met.

The aforementioned policy decisions provided a regulatory framework that focused on FDA regulatory authority, as well as the authority and responsibilities of the Centers for Medicare and Medicaid Service (CMS), over COVID-19 testing in CLIA-certified laboratories (Fig. 2; Table 1).

**Table 1.** Clinical laboratory improvement amendments (CLIA) [14]

Agencies involved	Food and Drug Administration (FDA)	Center for Medicare and Medicaid Services (CMS)	Centers for Disease Control and Prevention (CDC)
Responsibilities	Determines test complexity	Issues laboratory certificates	Provides analysis, research, and technical assistance
	Reviews waivers	Collects user fees	Develops technical standards and laboratory practice guidelines
	Develops rules for test complexity categorization	Conducts inspections and enforces compliance	Conducts quality improvement studies
		Approves private accreditation organizations, and state exemptions	Monitors proficiency testing practices
		Monitors laboratory performance	Develops and distributes information and educational resources
		Publishes CLIA rules and regulations	Manages the Clinical Laboratory Improvement Advisory Committee (CLIAC)

The primary responsibility of the FDA for COVID-19 testing is ensuring that *in vitro* diagnostics for the commercial market are safe and effective and that these diagnostics are appropriately manufactured, marketed, sold, and distributed. CMS is the primary agency for regulating CLIA statutory requirements for LDTs [14]. CLIA-certified laboratories must validate accuracy, precision, reportable ranges, and reference intervals for the laboratory’s patient population prior to reporting patient results for commercially available diagnostics and must also include validation of analytical sensitivity and specificity for LDTs.

## Streamlining of Laboratory Developed Test Practices to Address the COVID-19 Challenge

Out of necessity, academic and other clinical laboratories developed a number of innovations to meet the clinical demands of COVID-19 testing. These innovations were required due to the lack of testing capacity in the local clinical environment and the lack of an effective national testing approach. The following examples provide illustrations of team science approaches used to create LDTs for the COVID-19 pandemic.

**Fig. 3.**
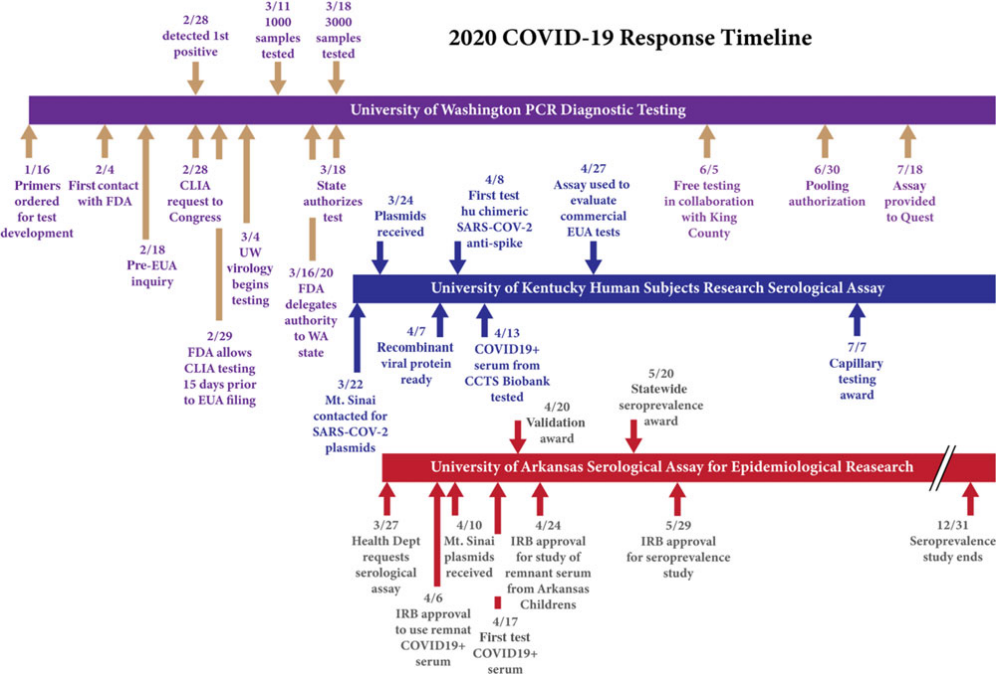
Timeline of laboratory responses for three Clinical and Translational Science Awards institutions. CCTS, Center for Clinical and Translational Science; CLIA, clinical laboratory improvement amendments; EUA, emergency use authorization; FDA, US Food and Drug Administration; IRB, Institutional Review Board; UW, University of Washington; WA, Washington.

As one of the first academic health care organizations to address the pandemic, the University of Washington (UW) drove engagement early and throughout the pandemic with the FDA, CDC, Washington State Department of Health, and professional societies. UW began COVID-19 test development in mid-January by obtaining the required reagents and supplies and conducting early validation studies (Fig. 3) [15, 16]. However, the nationwide lack of BSL-2-compatible positive control material for validation seriously hampered the institution’s ability to obtain authorization for COVID-19 testing. UW actively engaged with Congress to allow clinical laboratories to perform SARS-CoV-2 LDTs under CLIA *prior* to FDA authorization, a policy that was codified on February 29, 2020. When positive cases arrived in Seattle in the last week of February, UW investigators had the positive control material and regulatory authority to finish validation studies over one weekend and to begin testing the first week of March. In the first two weeks of operation, UW revalidated their RT-PCR assay multiple times, introducing high-throughput RNA extraction in order to process rapidly increasing sample numbers, and developed and incorporated alternate primer/probe sets as a response to supply chain shortages. The rapidly changing numbers and dynamic supply chain for SARS-CoV-2 testing required constant vigilance and revalidation of tests [17–19], resulting in the need for an equally rapid response from regulators. After the state of New York was granted the ability to authorize tests by the HHS, the state of Washington was granted similar authority on March 17, 2020. Notably, these two states also manage their own CLIA regulation and have proficient laboratory quality assurance departments with the capacity for this type of work. Local control of testing regulation allowed rapid feedback and responses (e.g., within 24 hours). This iterative, consultative process enabled rapid adaptation to changing FDA guidelines. For instance, UW began four-way pooling of COVID-19 PCR tests on June 29, 2020, days after the release of FDA guidance and three weeks before the first FDA authorization for pooling was granted [20]. Pooling enabled UW to continue to perform thousands of tests per day as reagent supply chains were reallocated to address the rise of COVID-19 cases in the southeastern and southwestern United States. To address anticipated shortages in RNA extraction supply chains, UW validated and published extraction-free testing in April of 2020 [21]. The early appearance of COVID-19 cases in the Seattle area, and its attendant publicity, pushed UW and the state to move swiftly and early in testing development and in the implementation of public health measures to attempt to control the spread of the pandemic.

At the University of Kentucky (UK), a multidisciplinary group was established to support the treatment, understanding, and eradication of COVID-19. This alliance, known as the University of Kentucky CURE Alliance (*C*OVID-19 *U*nified *R*esearch *E*xperts), included experts in virology, infectious disease, epidemiology, pharmacy, lung physiology, respiratory disorders, and clinical trials. The CTSA-supported UK Center for Clinical and Translational Science (CCTS) served as a CURE Alliance partner and leveraged its broad reach and established programs to provide coordination to the endeavor. Other partners included the Markey Cancer Center, UK Healthcare Clinical Laboratory, and the Department of Internal Medicine, Divisions of Infectious Disease, Pulmonary Medicine, Sleep Medicine, and Critical Care. Jointly this group established a COVID-19 biobank and launched a COVID-19 pilot grant program. The biobank collected samples from patients, which stimulated numerous research collaborations, as well as the serological studies described further.

The Biomarker Analysis Laboratory of the CCTS joined a CURE-sponsored team of basic science cores (Center for Molecular Medicine Protein Core, Flow Cytometry & Immune Monitoring Core, College of Pharmacy) to implement a serologic assay for COVID-19 antibodies. This group of basic science core laboratories cooperated to produce large quantities of high-quality viral antigens, which were used to reproduce the robust serological assay originally developed by Florian Krammer, Ph.D., Icahn School of Medicine at Mount Sinai (Fig. 3) [22]. Samples for protocol validation were provided by the CCTS-supported biobank. Subsequently, the developed serologic assay was used by the hospital clinical laboratory to validate commercial serological assays. This team science-enabled approach involved CCTS collaboration, the support of three basic laboratories within two colleges, and a third collaboration with the clinical laboratory. As a result, a new laboratory assay for COVID-19 was implemented in a little over a month. For example, cDNAs received from the Krammer laboratory on March 23 were used to develop an assay that supported the validation of commercial tests on April 27. Collectively, these efforts supported the development of new epidemiologic studies and created a new core laboratory for serology assays. In addition, a new diagnostic laboratory test using capillary sampling is in development. The new test, which uses volumetric absorptive micro-sampling, will provide a portable test for use in rural settings, allowing the CCTS to expand COVID-19 testing to rural counties in Kentucky.

The CTSA-supported Translational Research Institute (TRI) at the University of Arkansas for Medical Sciences (UAMS) worked with academic partners across departments and colleges and the state department of health to implement population sampling for COVID-19 seroprevalence testing. This work initially began as a collaboration among institutional virologists and a clinician scientist who jointly developed a four-antigen ELISA immunoassay (Fig. 3). This team adapted the ELISA protocol for detecting antibodies to SARS-CoV2 from the Krammer laboratory (described earlier) [23]. The Krammer laboratory shared test reagents to expedite reproduction of the assay. TRI worked with the basic immunology laboratory team, clinical pathology, informaticists, statisticians, infectious disease experts, and institutional and state public health officials to develop a state-supported seroprevalence study to monitor COVID-19 progression throughout the state. The study incorporates comparison testing of the four-antigen ELISA immunoassay with a EUA-approved SARS-CoV-2 antibody immunoassay in clinical use in the UAMS Clinical Laboratory. The incorporation of an analytical toxicologist with expertise in high-throughput clinical testing and robotics has provided a pathway for possible adoption of the four-antigen immunoassay by the clinical laboratory in the future, as well as an additional analytical research resource to the institution and the state for future assays. TRI worked closely with local institutional partners and regional campus partners to provide the regulatory infrastructure, statistics, data management, and sample collection resources to enable the collection of 9000 samples (7500 in adults and 1500 in children) over a six-month time period. In addition, a longitudinal study among college students was supported through the work.

Other collaborations supported diagnostic testing for COVID-19 throughout Arkansas. The UAMS Clinical Laboratory utilized five different platforms for RT-PCR diagnostic testing, including two with EUA approval and three LDTs based on the CDC assay, which were submitted for EUA approval at the time of development. The laboratory reduced other molecular diagnostic tests performed on existing instruments to meet clinical COVID-19 testing demands. Despite these efforts, the clinical laboratory could not support all of the testing needed by the community and relied on local, regional, and national referral laboratories to perform PCR testing, especially during the early months of the pandemic. Microbiologists worked with hospital clinical laboratory personnel to establish additional laboratory space and provide laboratory personnel for PCR testing for SARS-CoV2. While basic scientists were highly experienced with PCR, clinical testing required strict, unfamiliar approaches, including the maintenance and certification of their current instruments for clinical use, temperature monitoring, quality control, and meticulous documentation. Clinical pathologists worked with basic scientists to ensure that laboratory personnel training, instrument validation, and testing protocols met CLIA and College of American Pathologists (CAP) standards. Similarly, expertise at the Forensic DNA Section of the Arkansas State Crime Laboratory was used to support COVID-19 PCR testing and training of laboratory personnel at the Arkansas Department of Health – Public Health Laboratory. Finally, the UAMS Medical Center played a major leadership role for the state by establishing a drive-thru community test collection center and providing a van to increase drive-thru specimen collection opportunities throughout the state of Arkansas.

Thus, across multiple sectors, ranging from internal academic collaborations to external state collaborations, new teams were developed to meet the diagnostic testing challenges posed by the pandemic.

## Key Lessons Learned

Collectively, the experiences that supported effective responses to COVID-19 testing were influenced by the strength of existing relationships and collaborations. New collaborations were formed in order to pool both resources and expertise to rapidly validate COVID-19 testing platforms for the community. New collaborations also required education across academic and state-based institutions and departments. The combined expertise from these organizations provided relevant regulatory guidance for each institution. In Arkansas, it became obvious that a collaboration between clinical pathology and basic research laboratories was essential for COVID-19 PCR and antibody testing in order to maintain compliance with CLIA and accrediting organization standards for each lab performing COVID-19 PCR testing and to provide remnant samples needed for antibody tests. As a result, workforce morale improved and sample acquisition rates exceeded expectations. These collaborations continue to provide new resources and expertise for the research community that will bolster clinical and translational research and can be leveraged for future pandemics.

For UW, the pandemic showed the critical role that clinical laboratories and reference laboratories play in public health preparedness and response. The public health laboratory system runs less than 10% of tests and does not routinely perform high-volume testing on a daily basis. The COVID-19 pandemic also demonstrated the need for a federal and/or central system with the capacity to rapidly distribute BSL-2-compatible positive control materials for novel agents to test developers, whether at large commercial diagnostic companies or smaller clinical laboratories. Given that most pandemics are likely to start abroad, developing international networks for sample sharing is critical. For both the Ebola virus and COVID-19, Americans infected abroad have been repatriated to national quarantine units at the University of Nebraska Medical Center or on military bases. Specimens obtained from individuals quarantined at these units could provide important human samples for test development and validation in the early stages of future pandemics.

The high cost of early response to COVID-19 was initially covered by departmental reserves at the UW, but the approximately $30 million spent by the department between March and April 2020 represented an unsustainable expenditure that required external support. While UW was eventually able to obtain financial support from state and federal sources, the high cost of an effective pandemic testing response will continue to be a barrier for most entities seeking to rapidly expand their testing capacity. Beyond cost, it was also immediately apparent that the supplies, instrumentation, workers, and space required to mount an effective testing response would be difficult to sustain due to shortages or other pandemic-associated limitations. The lesson learned at UW, therefore, became “buy early, and buy a lot,” an approach obviously informed by the early impact COVID-19 had in this region.

One key challenge faced by states and academic health organizations during the first six months of the pandemic was the rapidly changing landscape around guidance for laboratory testing. The clearest example of this was the early declaration by the FDA asserting regulatory authority of LDTs, which required laboratories to submit EUA requests to the FDA. For many laboratories, this was an unfamiliar process, which caused both confusion and delay in test approval. While this changed with the August communication from HHS revoking such authority, it remains to be seen if these decisions will become policy for future pandemics.

## Recommendations for the Future

The aforementioned examples provided our institutions with experience that can be leveraged locally and nationally to improve pandemic-associated laboratory testing in the future. The following recommendations are offered for consideration as potential strategies that could improve federal and state-based responses to future pandemics.

1. Targeted funds should be available to public health laboratories and academic institutions to support the rapid validation of high-throughput testing platforms.
2. A coordinated laboratory response system should be made available that would allow for the rapid distribution of BSL-2-compatible positive control materials for novel agents to test developers.
3. Given that future pandemics will likely start outside the United States, international laboratory networks for sample sharing should be established.
4. Public–private partnership networks should be established that could be readily activated during future pandemics to overcome supply chain barriers, including preanalytical (swabs, transport media, pipette tips, etc.) and analytical supplies (media, etc.).
5. COVID-19 related partnerships among CTSAs, academic laboratories, state public health laboratories, and other state-based institutions should be maintained to expedite responses to future pandemics.
6. Designated funds to support the standardization of the now numerous SARS-CoV-2 tests should be provided to the Food and Drug Administration, as well as the future allocation of funds in this area for future pandemics.
7. Thorough assessment of the EUA process and modernization of the process to maximize efficiency during pandemics [4].

## Conclusion

Rapid employment of diagnostic testing for COVID-19 has been a key component of the public health response to the pandemic. Out of necessity, academic and other clinical laboratories worked together to develop a number of laboratory testing innovations to meet the clinical demands of COVID-19 testing. These collaborations may not have been possible outside of the pandemic, and it became evident that without these collaborations, testing practices and lagging test results may have suffered all the more. Based on the experiences of several CTSA institutions across the United States, the development of laboratory-developed tests during the COVID-19 pandemic can inform improved laboratory responsiveness to future pandemics and provide new opportunities to apply team science approaches in clinical and translational research.
